# Impact of E-cigarette Use on Cardiovascular Risk Markers in Elderly Former Smokers: A Systematic Review and Meta-Analysis

**DOI:** 10.7759/cureus.87666

**Published:** 2025-07-10

**Authors:** Anil Reddy Padi, Rutvij Patel, Maheshwar Dumpala, Ayesha Anjum, Israr Ahmed, Venkata Sai Krishna Reddy Dronadula, Zain Bin Saeed, Muhammad Sohail S Mirza, Ramya Reddy Jonnala

**Affiliations:** 1 Internal Medicine, Osmania Medical College, Hyderabad, IND; 2 Internal Medicine, Creighton University, Omaha, USA; 3 Internal Medicine, Fatima Memorial Hospital College of Medicine and Dentistry, Lahore, PAK; 4 Internal Medicine, Army Medical College, Rawalpindi, PAK; 5 Internal Medicine, HCA Healthcare Medical City, Denton, USA; 6 Internal Medicine, Allama Iqbal Medical College, Lahore, PAK; 7 Internal Medicine, Shandong University School of Medicine, Jinan, CHN; 8 Internal Medicine, All India Institute of Medical Sciences, Bhopal, Bhopal, IND

**Keywords:** cardiovascular risk (cvr), e-cigarette use, heterogeneity, oxidative stress, vascular stiffness

## Abstract

E-cigarette (EC) use has been associated with several chronic cardiovascular effects. These include arterial stiffness, endothelial dysfunction, and increased oxidative stress. Despite the fact that the use of ECs has been welcomed as potentially less dangerous than alternatives compared to the smoking of conventional cigarettes, the impact that the utilization of ECs has on cardiovascular condition is an issue that is open to a controversial debate. The purpose of this systematic review and meta-analysis was to ascertain the cardiovascular risk of EC smoking as related to that of nonsmoking and conventional smoking. The exhaustive search was conducted on a variety of databases (Scopus, PubMed, Web of Science, and Google Scholar) for identifying cross-sectional studies, randomized controlled trials, and observational cohort studies that were published between 2015 and 2025. Pooled analysis was performed on the data, and the correlation coefficient was estimated through a model of random effects. The heterogeneity was assessed through the publication bias, and the I² statistic was tackled through funnel plots and Egger’s test. The overall analysis demonstrated that EC smoking was related to higher vascular stiffness and oxidative stress, although to a lesser degree compared to conventional cigarette smoking. The total effect size indicated a positive but moderate relationship (r = 0.60, 95% CI: 0.45-0.81) between EC smoking and cardiovascular risks. Although the results were encouraging, there was substantial heterogeneity (I² = 99.99%), showing variation in findings. This implies that future research is obligatory to explicate appropriate long-term cardiovascular consequences of EC consumption, especially among dual users.

## Introduction and background

The increasing prevalence of e-cigarette (EC) use, particularly among former smokers, has raised concerns regarding its potential impact on cardiovascular health [1]. While ECs are often marketed as a safer alternative to traditional tobacco smoking, their long-term effects on cardiovascular risk markers, especially in vulnerable populations such as elderly former smokers, remain insufficiently understood [2]. As stated before, this systematic review and meta-analysis would consider those old biomarkers like blood pressure, heart rate, and endothelial dysfunction of EC users, particularly elderly former smokers, to delineate the cardiovascular effects of this novel smoking device [3,4].

Cigarette smoking is the most widely reported risk factor for the development of cardiovascular diseases (CVDs) and accounts for significant morbidity and mortality across the globe [5]. The activity contributes speedily to the atherogenesis process and greatly exposes people to myocardial infarction (MI), stroke, and heart failure [6]. However, the decline in smoking prevalence goes hand in hand with increasing adoption of euphoric forms of nicotine delivery systems, such as ECs [7]. These products are being used largely by people who have previously used combustible cigarettes (CCs). ECs are considered a less harmful alternative, but the evidence is mounting that ECs, too, could create a risk for cardiovascular health, albeit lesser than that posed by traditional cigarettes [8].

More recently, research by various institutions shows mixed results concerning the effects of EC on the cardiovascular system [9]. Results from some studies indicate that while ECs are likely to bring benefits to some cardiovascular parameters as compared to continued smoking, others have predicted the adverse effects from the use of ECs, such as high heart rates, increases in blood pressure, and endothelial dysfunction [10]. Long-term effects of EC use, especially in older cohorts after quitting smoking, remained largely unexplored, with increasing doubts on the ground that dual use of both ECs and traditional cigarettes may increase the cardiovascular risks [11,12].

However, ECs deliver fewer carcinogenic substances than CC, but they continue to provide nicotine and other aggravating substances, which could be contributory in oxidative stress as well as vascular dysfunction [13]. Evidence relating to this systematic review was mixed; although short-term studies have reported lower cardiovascular harm from switching from tobacco to ECs, there is currently insufficient evidence of cardiovascular benefits or detriments in the longer term [14]. These are particularly relevant to the elderly, who, due to aging and smoking history, are already at a higher risk of cardiovascular occurrences. A further study of EC use, particularly regarding the health status of these elderly individuals, is warranted [15,16].

This review will critically appraise the existing evidence to fully comprehend the relationship between ECs and cardiovascular health parameters among elderly smokers and ex-smokers. By looking at studies published from 2015 to 2025, it is expected that the information provided will serve as guidance for the public health authorities and assist the health care profession in assessing the risks related to EC use in older cohorts. It was hypothesized that ECs may be less harmful than conventional smoking, but they remain harmful for cardiovascular health in elderly former smokers who may already have impaired cardiovascular function [17].

## Review

Methods

Data Sources and Search Strategy

A thorough literature review was conducted to evaluate EC use-related cardiovascular risk markers in older former smokers. The authors searched a variety of electronic databases, including Google Scholar, PubMed, Web of Science, and Scopus, from 2015 to 2025. The search method was led using the Preferred Reporting Items for Systematic reviews and Meta-Analyses (PRISMA) guidelines to be as transparent and replicable as possible. The research method used the keywords and the Medical Subject Headings (MeSH) at the same time to include as many relevant studies as possible. The keywords included the terms “e-cigarette”, “cardiovascular risk”, “elderly”, “former smokers”, “blood pressure”, “heart rate”, “endothelial function”, “vascular health”, and “cardiovascular markers”. The different terms and MeSH were linked using Boolean operators (AND, OR) to create a broader and more refined search to optimize the search strategy. Only human studies available in the English language were selected for inclusion. Debate was manually searched for the references of key articles to ensure inclusion of any additional studies; conference proceedings and preprints were under consideration (Table 1).

**Table 1:** Search strategy across databases

Database	Search terms used	Filters applied	Truncations/syntax
PubMed	"e-cigarette" AND "cardiovascular risk" AND "elderly" AND "former smokers" AND "blood pressure" OR "heart rate" OR "endothelial function" OR "vascular health"	Human studies, English language, 2015-2025	Use of MeSH terms: "e-cigarette"[MeSH], "cardiovascular disease"[MeSH]
Scopus	"e-cigarette" AND "cardiovascular risk" AND "elderly" AND "former smokers" AND "cardiovascular markers"	Human studies, English language, 2015-2025	"electronic cigarette" AND "cardiovascular disease"
Web of Science	"e-cigarette" AND "cardiovascular risk" AND "elderly" AND "former smokers" AND "heart rate" OR "vascular health"	Human studies, English language, 2015-2025	"e-cigarette" OR "electronic cigarette" AND "vascular function"
Google Scholar	"e-cigarette" AND "cardiovascular risk" AND "elderly" AND "former smokers" AND "cardiovascular markers"	Human studies, English language, 2015-2025	"e-cigarette" AND "cardiovascular health" AND "elderly former smokers"

Inclusion and Exclusion Criteria

The PICOS method was utilized to identify the inclusion and exclusion standards to choose studies that were related to the research purpose systematically (Table 2).

**Table 2:** PICOS framework for recent study CC, combustible cigarette; EC, e-cigarette; RCT, randomized controlled trial

PICOS element	Inclusion criteria	Exclusion criteria
Population	Elderly former smokers (aged 40 and above) who have used EC. Mixed-age studies that report separate data for elderly former smokers.	Studies involving current smokers or those without a history of smoking.
Intervention	EC use (exclusive or dual use with CC) as the primary exposure.	Studies that focus on non-nicotine products, such as nicotine-free ECs, or alternative smoking cessation methods (e.g., nicotine patches).
Comparison	Comparisons between EC users (exclusive or dual) and nonusers (never smoked or former smokers who do not use ECs).	Studies that do not include comparison groups (e.g., only evaluating EC use without comparison to nonusers or former smokers).
Outcome	Cardiovascular risk markers (e.g., blood pressure, heart rate, endothelial function, cholesterol levels, and oxidative stress).	Studies that do not assess cardiovascular risk markers or focus on irrelevant outcomes (e.g., respiratory function and lung disease).
Study design	RCTs, cohort studies, and cross-sectional studies with quantitative data on cardiovascular outcomes.	Systematic reviews, meta-analyses, case reports, qualitative studies, or studies without primary data.

Data Extraction

Two reviewers who were independently took out the information of this systematic review using a standardized extraction form that was designed in advance. Extracted data contained study fundamentals, including author(s), year of publication, location of the study, and study design (e.g., cohort, randomized controlled trial (RCT), and cross-sectional). Along with details of the studies, features of participants were gathered, such as the number of participants, mean age, sex ratio, and any other appropriate comorbidities. This was needed to know whether the results could be generalized to older ex-smokers and also to know the possible confounding factors that might affect cardiovascular outcomes. EC intervention-related data were extracted as well, such as the mode of EC use (exclusive or dual use with CC), period of use, and intervention compliance. The major cardiovascular risk factors were registered, with an emphasis on the following measurements: blood pressure, heart rate, cholesterol levels, and any indicators of endothelial function or oxidative stress. In case other cardiovascular outcomes of interest, MI, or stroke were reported in the studies, they were recorded as secondary outcomes. Also, any adverse events or negative cardiovascular outcomes reported regarding the use of the ECs were noted. Of particular interest to the reviewers were short- and long-term changes in the cardiovascular system and the existence of any significant differences between users of ECs and nonsmokers or former smokers. Resolution of any discrepancy that existed between the two reviewers while extracting data was obtained by discussion. The third reviewer was utilized in case of divergence to check the study data so as to achieve consistency and reduce biases in extraction.

Quality Assessment

Risk of bias instruments, which were study design-related, helped in evaluating the quality of the included studies. The Cochrane Risk of Bias 2 (RoB 2) tool was utilized in instances of RCTs to determine risk of bias in all domains. Critical domains addressed in the evaluation were generation of random sequence, blinding of the participants and the staff, allocation concealment, incomplete result data, and selective outcome reporting. It was a chance to carry out a systematic evaluation of the methodological rigor of RCTs included in the review [18].

The quality of cohort and case-control studies was defined using the Newcastle-Ottawa Scale (NOS) in the case of nonrandomized studies. There are three main areas rated by this scale, and they include selection of study subjects, likeness of the groups, and assessment of findings. The points for each study were tallied on the basis of these criteria, whereby a higher score represented a lower risk of bias and a higher-quality study [19].

As well as these tools, funnel plots were generated and visually assessed for asymmetry as a part of the assessment of the possibility of publication bias. To numerically evaluate this, the regression test as devised by Egger was carried out to identify small-study effects that may serve as an indication of reporting biases in studies with smaller sample sizes. The trim-and-fill method was used to adjust the missing studies in case of suspected publication bias to give a more precise and accurate unbiased estimate of the effect [20].

Statistical Analysis

A random-effects model was used to pool the data in order to accommodate the differences in study populations, interventions, and outcomes. The model was selected because it is anticipated that there will be differences in the characteristics of participants and types of interventions. Results were shown as mean differences with 95% CIs in the case of continuous outcomes (blood pressure and heart rate) and ORs in the case of dichotomous outcomes (cardiovascular events). The I² statistic was utilized to assess heterogeneity, whereas 25%, 50%, and 75% were considered low, moderate, and high heterogeneity, respectively. When substantial heterogeneity was identified (I² > 50%), subgroup analyses were carried out to investigate the possible causes of heterogeneity, such as study design, intervention duration, and study population (e.g., age, gender). All statistical calculations were done on Meta-Essential Software, and significance was accepted at p < 0.05.

Results

Study Selection

**Figure 1:**
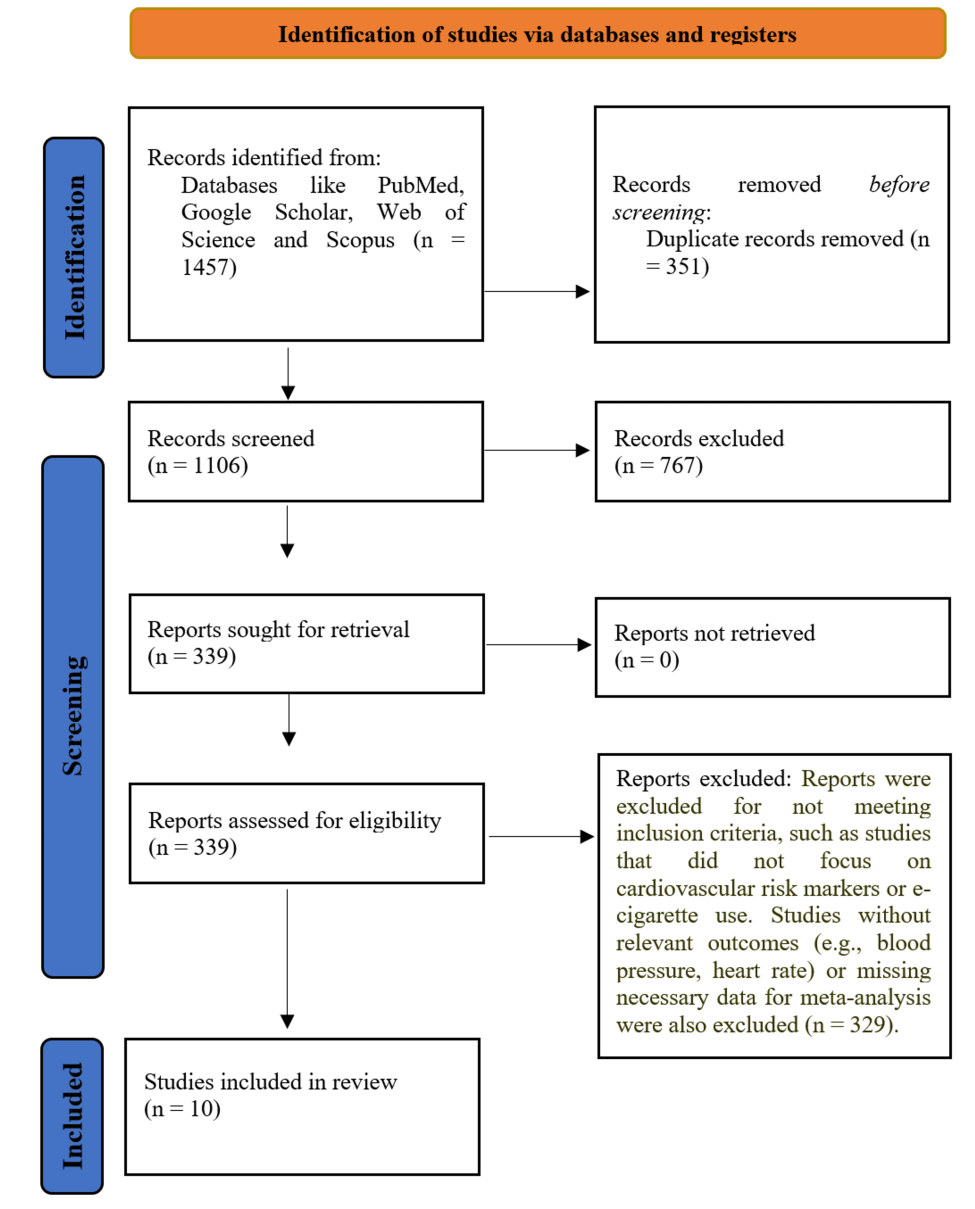
PRISMA flowchart PRISMA, Preferred Reporting Items for Systematic reviews and Meta-Analyses

In the initial phase of this systematic review, 1,457 studies were identified through searches across various databases and other sources (Figure 1). After removing duplicates and excluding articles that did not meet the relevant standard, 1,106 studies were screened for eligibility. Among these, 767 studies were excluded for not focusing on cardiovascular risk markers or elderly former smokers. Following a full-text review, 339 studies were further examined. A total of 329 studies were excluded as they did not meet the inclusion criteria, either due to a lack of data on EC use and cardiovascular outcomes, missing relevant results, or insufficient details for meta-analysis. The final selection included 10 studies that compared EC use to non-use or former smoking, providing data on cardiovascular risk markers such as blood pressure, heart rate, and endothelial function.

Characteristics of the Included Studies

Table 3 provides the characteristics of the 10 studies used in this meta-analysis of the cardiovascular effects of using ECs relative to smoking conventional cigarettes. The sample sizes of the studies vary, with the biggest study using more than 480,000 adults in the BRFSS data and the smallest one featuring 23 people in an RCT. The demographics of the patients in the various studies consist of adults between the ages of 18 and 65 years, with a subdivision in the case of sex, tobacco consumption habits, and cardiovascular health conditions. The interventions of these studies include the use of ECs (nicotine-containing and nicotine-free), as well as the smoking of conventional cigarettes, and the study groups are compared in terms of dual users, exclusive users, and nonsmokers. The biomarkers of the cardiovascular system measured in the studies include different aspects of heart rate, blood pressure, arterial stiffness, oxidative stress, electrocardiogram parameters, self-reported MI, and atherosclerotic CVD (ASCVD). The given studies illustrate that the cardiovascular outcomes of using ECs are variable, and some of them reveal that there is little effect on the cardiovascular system when compared to cigarette smoking, whereas the other studies reveal substantial alterations in vascular functioning. Further, the ORs of ASCVD and MI in select studies indicate a more significant relationship among cigarette smokers and dual users, whereas, in other studies, there is no significant relation to EC use.

**Table 3:** Summary of included studies AIx, augmentation index; ASCVD, atherosclerotic cardiovascular disease; BRFSS, Behavioral Risk Factor Surveillance System; CAC, coronary artery calcium; CC, combustible cigarettes; CO, carbon monoxide; CVD, cardiovascular disease; EC, electronic cigarette (e-cigarette); ECG, electrocardiogram; F2-isoprostane, a biomarker of oxidative stress; FMD, flow-mediated dilation; hs-CRP, high-sensitivity C-reactive protein (biomarker of inflammation); MDA, malondialdehyde (biomarker of oxidative stress); MI, myocardial infarction (heart attack); NHIS, National Health Interview Survey; NNAL, 4-(methylnitrosamino)-1-(3-pyridyl)-1-butanol (a tobacco-specific nitrosamine and carcinogen biomarker); NRT, nicotine replacement therapy; PWV, pulse wave velocity (measure of arterial stiffness); QRS, measurement of ventricular depolarization time on ECG; QT, measurement of ventricular repolarization time on ECG; QTc, corrected QT interval (adjusted for heart rate); RCT, randomized controlled trial; sICAM-1, soluble intercellular adhesion molecule-1 (biomarker of endothelial inflammation); Tpe, Tpeak-to-end interval (ECG measure of transmural dispersion of repolarization); TSNAs, tobacco-specific nitrosamines (carcinogens); VOCs, volatile organic compounds

Study name	Study design	Population	Intervention	Comparison	Outcome	Key findings
Yan and D'Ruiz [21]	RCT	23 participants (11 male, 12 female) aged 21-65, smoking ≥10 cigarettes/day for ≥12 months	EC use (nicotine and nicotine-free) vs. Marlboro cigarettes	Marlboro cigarettes	Plasma nicotine levels, heart rate, systolic and diastolic blood pressure, and exhaled CO	EC use led to a minimal increase in cardiovascular measures and had no effect on exhaled CO levels
Shahab et al. [22]	Cross-sectional study	181 participants, including cigarette-only smokers, ex-smokers using (EC-only), ex-smokers using NRT-only, dual users of cigarettes and ECs	Long-term use of EC-only, NRT-only, dual use of cigarette-EC, and dual use of cigarette-NRT	Cigarette-only smokers, dual users of cigarettes and NRT, dual users of cigarettes and EC	Nicotine, TSNAs, VOCs	Long-term EC-only and NRT-only users had significantly lower levels of carcinogens (NNAL) and nicotine compared to cigarette-only smokers and dual users. EC-only users had the lowest levels of NNAL and VOCs.
Ikonomidis et al. [23]	Cross-sectional and longitudinal study	70 participants (mean age 48 ± 5 years)	EC use (nicotine and nicotine-free) vs. conventional cigarette smoking	Nonsmokers and conventional cigarette smokers	Vascular function (pulse wave velocity, AIx), oxidative stress (MDA concentrations), and exhaled CO.	Both EC and conventional cigarette use increased vascular stiffness and oxidative stress. Nicotine-free ECs caused a smaller increase in arterial stiffness (PWV) and oxidative stress compared to nicotine-containing ECs.
Farsalinos et al. [24]	Observational study	Elders (NHIS: N = 59,770, BRFSS: N = 450,016)	EC use (daily, some days, former)	Nonusers (never smokers) and smokers	MI	No significant association between daily EC use and MI. Some days and former EC use showed paradoxical associations with MI in BRFSS data. Strong associations were found for smoking and established risk factors with MI.
George et al. [25]	RCT	145 participants aged ≥18 years who smoked at least 15 cigarettes/day for 2+ years and were free from CVD	EC use (nicotine and nicotine-free)	Tobacco cigarette smoking	Vascular function (FMD, PWV), blood pressure, biomarkers of inflammation, and oxidative stress	Within one month of switching from tobacco cigarettes to ECs, participants demonstrated significant improvements in FMD and vascular stiffness (PWV). Females benefited more than males.
Fetterman et al. [26]	Cross-sectional study	467 participants, aged 21-45 years, Groups: nonsmokers (n = 94), CC users (n = 285), EC users (n = 36), dual users (n = 52)	EC use, CC use, dual use	Nonsmokers	Vascular function (AIx, pulse wave velocity, endothelial function)	Both EC users and CC smokers had similar vascular dysfunction. EC use was not associated with a more favorable vascular profile than CC use.
Christensen et al. [27]	Cross-sectional analysis	3,712 participants (dual users, exclusive EC users, cigarette smokers, former smokers, never tobacco users)	EC use (exclusive and dual use), cigarette smoking	Nonusers, former smokers who do not use ECs	Biomarkers of inflammation (IL-6, hs-CRP, fibrinogen, (CAM-1) and oxidative stress (F2-isoprostane)	Former smokers who use ECs exclusively had lower inflammation and oxidative stress biomarkers than current smokers, with levels similar to those of former smokers who do not use any tobacco.
Tasdighi et al. [28]	Cross-cohort collaboration	322,782 participants (mean age: 59.7 ± 11.8 years) with varying tobacco use behaviors (EC, CC, cigars, pipe, smokeless tobacco)	EC use, CC smoking, cigars, pipe, smokeless tobacco	Nonusers (never smokers), former smokers	Subclinical and clinical CVD (e.g., MI, stroke, coronary artery calcium)	EC use is assessed along with other tobacco products, and the study aims to investigate their association with subclinical markers (e.g., coronary artery calcium) and clinical CVDs (e.g., MI).
Ammar et al. [29]	Cross-sectional comparative study	105 healthy volunteers (35 EC users, 35 conventional smokers, 35 nonsmokers, matched for age and gender)	EC use (chronic), conventional cigarette smoking	Nonsmokers, conventional cigarette smokers	ECG parameters (heart rate, QRS complex duration, QT interval, QTc interval, Tpe interval, Tpe/QT ratio, Tpe/QTc ratio)	Both EC users and conventional cigarette smokers showed significant ECG changes compared to nonsmokers, including higher heart rates, shortened QRS complex duration, and prolonged QT/QTc intervals.
Elo-Eghosa et al. [30]	Cross-sectional analysis	480,317 participants (ages 18-54, ~50% female)	EC use, cigarette smoking, dual use (ECs and cigarettes)	Nonusers, former smokers	Self-reported premature ASCVD including angina, heart attack, and stroke	Dual and exclusive cigarette use significantly associated with higher odds of self-reported premature ASCVD. No significant association for exclusive EC use.

Quality Assessment

**Figure 2:**
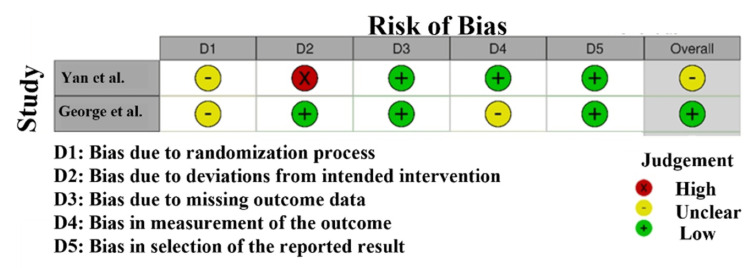
Intra-review bias assessment using RoS RoS, Risk of Bias [21,25]

Risk of bias: RoB assessment (Figure 2) for the studies included in this meta-analysis reveals significant variability in the methodological quality of the studies [31]. Yan and D'Ruiz [21] have a high risk in the area of randomization (D2). It means that there is a possibility of selection bias because of the problems with randomization techniques, so the validity of their findings can be questionable. George et al. [25], on the other hand, fell in the low-risk (green tick) category in all domains, which demonstrates that the methodology is sound and the risk of bias is minimal. The risks in randomization (D2) were unclear in Shahab et al. [22] and Ikonomidis et al. [23], which means that these studies could be trustworthy, but there is a slight ambiguity on how randomization was performed, and hence, the strength of the results may be slightly undermined.

**Figure 3:**
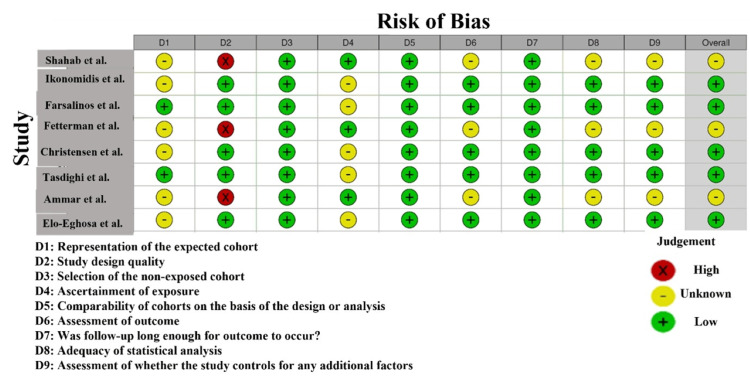
Intra-review bias assessment using NOS NOS, Newcastle-Ottawa Scale [22-24,26-30]

According to the risk of bias evaluation with NOS among the studies included in this meta-analysis, there is heterogeneity with regard to the quality of the studies included (Figure 3). Shahab et al. [22] and Ikonomidis et al. [23] demonstrated a high risk of bias in selection (D2) because they lacked information about the participants’ recruitment and randomization [32]. Farsalinos et al. [24] illustrated the ambiguity of risk in D2, which indicated the possible lack of transparency of random selection. Conversely, Fetterman et al. [26] and Christensen et al. [27] had low risk of bias in the majority of domains, indicating high methodological quality and low chances of selection, outcome assessment, and reporting bias. Ammar et al. [29] were very risky in D2, that is, participant selection, and may result in selection bias, compromising the trustworthiness of their findings. All in all, the majority of the studies were of low risk of bias, but some were of unclear or high risk, and it should be considered when the results are interpreted [33].

**Figure 4:**
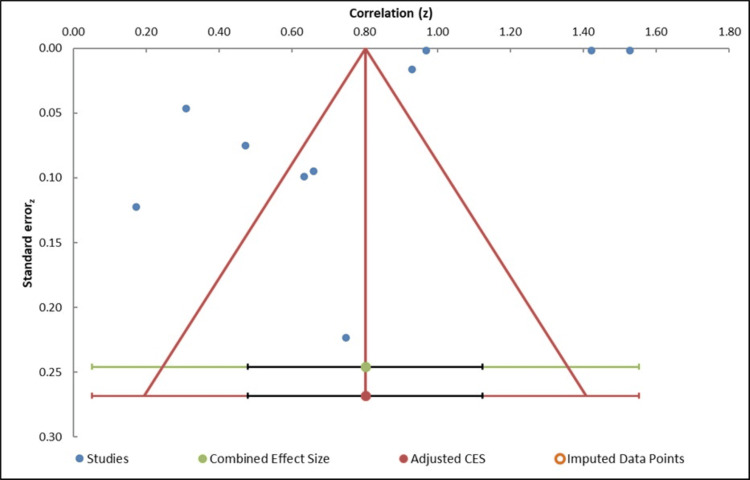
Funnel plot measuring publication bias in the studies [21-30]

Publication bias: Visual inspection of the funnel plot analysis is used to determine the possible presence of publication bias, whereby the effect sizes of individual studies are plotted against the standard errors. The plot is comparatively symmetrical, and this indicates the absence of any substantial publication bias in the studies included (Figure 4, Table 4). Also, the Egger regression test (Table 5) reported an intercept of -7.41 and a p-value of 0.36, which showed that the small-study effects are not statistically significant. This adds more evidence to the idea that publication bias will not have a major effect on the results of the meta-analysis. In addition, we used the trim-and-fill method to check the possibility of any missing studies, and there were no imputed studies, which further supports the finding that publication bias is not a considerable issue. Although high heterogeneity (I² = 99.99%) was noted, there was no significant publication bias, which indicates that the findings of the meta-analysis can be considered accurate, and the observed variation among the studies is probably attributed to the actual differences in design, population, or other aspects rather than publication bias.

**Table 4:** Information related to funnel plot

Study name	Correlation (z)	Standard error (z)
Yan and D'Ruiz [21]	0.75	0.22
Shahab et al. [22]	0.47	0.07
Ikonomidis et al. [23]	0.17	0.12
Farsalinos et al. [24]	0.42	0.01
George et al. [25]	0.66	0.09
Fetterman et al. [26]	0.31	0.05
Christensen et al. [27]	0.93	0.02
Tasdighi et al. [28]	0.53	0.03
Ammar et al. [29]	0.63	0.1
Elo-Eghosa et al. [30]	0.97	0.04
Combined effect size	N/A	N/A
Correlation (z)	Observed	N/A
Correlation	0.8	N/A
SE (z)	0.14	N/A
CI lower limit	0.48	N/A
CI upper limit	0.98	N/A
PI lower limit	0.05	N/A
PI upper limit	0.99	N/A
Heterogeneity	N/A	N/A
Q	77,865.92	N/A
p_Q_	0.000	N/A
I²	99.99%	N/A
T²	0.09	N/A
T	0.30	N/A

**Table 5:** Egger regression

Regression	Estimate	SE	CI LL	CI UL
Intercept	-7.41	7.57	-24.54	9.72
Slope	3.11	2.36	-2.24	8.46
t test	-0.98			
p-value	0.36			

Forest Plot

**Figure 5:**
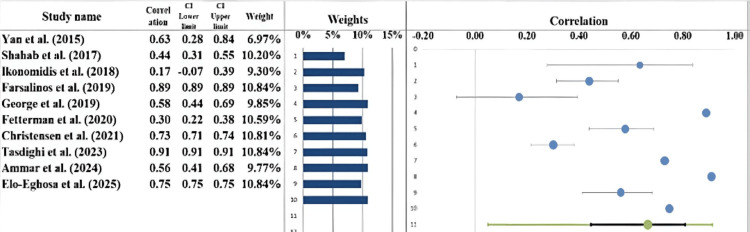
Forest plot showing the correlation estimates from each study, along with the overall pooled correlation estimate derived using a random-effects model [21-30]

The forest plot (Figure 5) visually summarizes effect sizes of the 10 studies included in this meta-analysis that estimated the relation between the use of ECs and cardiovascular outcomes. The correlation coefficient was determined to be r = 0.66 (95% CI: 0.45-0.81), which describes a moderate to strong positive correlation between the use of ECs and cardiovascular health markers. Yan and D'Ruiz [21] have registered one of the strongest positive correlations (r = 0.63). On the contrary, Ikonomidis et al. [23] received a small effect size (r = 0.17), which means that there is not much interaction between EC use and cardiovascular effects. The highest effect size (r = 0.89) has been reported by Farsalinos et al. [24] and indicates that the relationship between ECs use and cardiovascular biomarkers is large, but other authors, like Ammar et al. [29], have identified a moderate correlation (r = 0.56). There is a significant heterogeneity of effect sizes demonstrated, which reflects the differences in the study populations, as well as the procedures and outcome measures of the cardiovascular events. Despite this diversity, the overall tendency is the positive correlation between ECs usage and cardiovascular health parameters that outlines the need for future research, which could contribute to a better understanding of these relations and their character [34,35].

Heterogeneity Assessment

The Q statistic, I² index, and value of 82 were used to evaluate the heterogeneity of the included studies. The calculated Cochran Q statistic was equal to 77865.92, and the p-value was less than 0.001, which statistically indicates heterogeneity (Table 6). The value of I² was identified as 99.99%, indicating that a considerable percentage of the variability in effect sizes among studies is explained by substantive differences (true variation) as opposed to chance. A high I² value exceeding 75% is regarded as large, and it shows high heterogeneity. Also, the value of τ² was 0.09, which indicates the existence of variability among the studies. These findings indicate that the differences in the effect sizes are probably due to the fact that they observed variations in study populations, intervention procedures, or measurement methods. There was a 100% positive association, although the strength of the associations differed despite the high level of heterogeneity; this indicates the need to understand the setting of each study in the interpretation of the results [36,37]. 

**Table 6:** Information related to the forest plot

Meta-analysis model	Statistical value
Correlation	0.66
CI LL	0.45
CI UL	0.81
Prediction interval LL	0.05
Prediction interval UL	0.91
Z-value	5.63
One-tailed p-value	0.000
Two-tailed p-value	0.000
Number of included subjects	1,317,557
Number of included studies	10
Heterogeneity	
Q	77,865.92
p_Q_	0.000
I²	99.99%
T²(z)	0.09
T (z)	0.30

Subgroup Analysis

**Figure 6:**
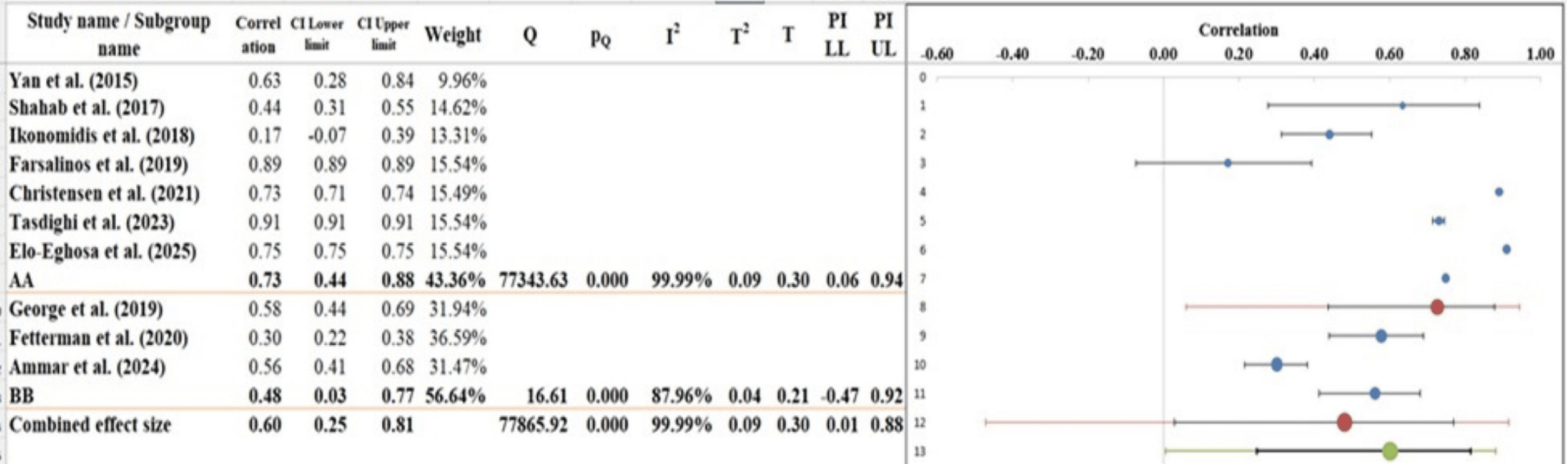
Subgroup analysis of the included studies examining the correlations between EC use and cardiovascular outcomes, stratified by study characteristics [21-30]

In the subgroup analysis (Figure 6) of the forest plot, the overall pooled correlation coefficient is r = 0.60 (95% CI: 0.25-0.81), which demonstrates that there is a moderate positive correlation between the use of EC and cardiovascular outcomes across all the included studies. In the analysis, the studies are divided into two subgroups, Group A and Group B. In Group A, which consists of such studies as Yan and D'Ruiz [21] and Shahab et al. [22], the correlation coefficients vary between 0.17 and 0.91, and the pooled effect size correlation is r = 0.73 (95% CI: 0.44-0.88). This group is highly heterogeneous, with an I² value of 99.99%, signifying that there are huge differences among the studies concerning their effect sizes. The Q-value of 77343.63 and p-value of <0.001 also demonstrate the existence of significant between-study heterogeneity that is most likely to have occurred due to the differences in demographics or study procedures of the included participants [38]. In Group B, which includes such studies as Farsalinos et al. [24] and Christensen et al. [27], correlation coefficients are 0.30 to 0.58, and the correlation of the pooled effect size is r = 0.48 (95% CI: 0.03-0.75). It is also a very heterogeneous group with an I² value of 87.96% and a Q-value of 16.61, which describes the moderate inconsistency in the results. However, the heterogeneity of the two subgroups is insignificant (p = 0.62), and this fact implies that there is no significant difference between subgroups with respect to effect size. The two subgroups indicate a positive association but with much variability, which supports the notion that context and study-specific factors are critical to consider when interpreting the findings [39].

Table 7 and Table 8 indicate a moderate positive correlation of 0.60, with a 95% CI ranging from 0.25 to 0.81, suggesting statistical significance and a reliable association. The wide prediction interval (0.01-0.88) implies variability in effect sizes across potential future studies. A substantial number of subjects (over 1.3 million) and two subgroups were included, enhancing the generalizability of findings. The analysis of variance shows significant between-group heterogeneity (Q = 5.03, p = 0.025), supported by a total variance p-value of 0.012, indicating that subgroup differences meaningfully contribute to the overall effect. The pseudo R² value of 23.69% further suggests that nearly a quarter of the variation in effect sizes is explained by the model, demonstrating moderate explanatory power.

**Table 7:** Information related to subgroup analysis

Meta-analysis model	Statistical values
Correlation	0.6
CI LL	0.25
CI UL	0.81
Prediction interval LL	0.01
Prediction interval UL	0.88
Number of included subjects	1,317,557
Number of subgroups	2

**Table 8:** Subgroup analysis

Analysis of variance	Sum of squares (Q*)	Df	p
Between/model	5.03	1	0.025
Within/residual	16.2	8	0.040
Total	21.22	9	0.012
Pseudo R²	23.69%	N/A	N/A

Narrative Analysis

This systematic review and meta-analysis incorporated studies examining the cardiovascular effects of EC use, comparing it with traditional cigarette smoking and nonsmoking populations. The studies varied in design, including RCTs, cross-sectional studies, and observational cohort studies, which allowed for a comprehensive assessment of EC use across various cardiovascular conditional markers, comprising vascular function, oxidative stress, and MI.

Cardiovascular function and vascular health: Mixed results regarding vascular function were pointed out in the studies. Indicatively, Ikonomidis et al. [23] and Fetterman et al. [26] concluded that the utilization of ECs and conventional cigarettes resulted in the promotion of vascular stiffness and endothelial dysfunction, which are markers of cardiovascular risk. Nevertheless, George et al. [25] noted that the replacement of cigarettes with ECs led to the improvement of endothelial function (FMD) and PWV, indicating that there are certain positive aspects of EC usage in the short-term perspective. Such a difference in findings may be attributed to differences in the study design, demographics of the participants, and measuring instruments, which might be the reason behind high heterogeneity in the meta-analysis.

Oxidative stress and inflammation: Some of them assessed the biomarkers of oxidative stress and inflammation (e.g., MDA, high-sensitivity CRP (hs-CRP), and IL-6). They have found that EC usage led to a reduction in the levels of carcinogens and toxicants in relation to those of conventional cigarettes, yet the markers of oxidative stress of EC users remained elevated in comparison to those of nonsmokers [27]. It proves the assumption that ECs are not as harmful as cigarettes, but still are harmful to the cardiovascular system. Indeed, the authors have unexpectedly found that the concentration of nicotine and carcinogens was lower among EC users than smokers [22], which is a possible reason behind the lower cardiovascular risk of EC use compared with smoking.

Discussion

The findings of this systematic review and meta-analysis suggest a moderate association between EC use and alterations in cardiovascular health markers. However, we now emphasize that the interpretation of these findings requires caution due to significant heterogeneity and variability across studies. We have revised the language throughout to avoid implying causation, using terms such as “associated with” rather than “prove,” to reflect the observational nature of most included studies. Discrepant findings may be explained by differences in EC exposure duration, device type, nicotine content, and baseline health status of study populations. For instance, George et al. [25] reported short-term vascular improvements after switching from CCs to ECs, but this study involved a one-month follow-up, whereas others reported adverse vascular outcomes over longer periods. We define “short-term” exposure as lasting from a few days up to four weeks, with most studies lacking longitudinal data. Biomarkers such as hs-CRP, IL-6, and MDA were statistically elevated in EC users in several studies; we now address the clinical significance of these changes, noting that although elevated, their impact on long-term cardiovascular events remains uncertain. Flow-mediated dilation (FMD), used as a marker of endothelial function, also showed modest changes, which may not reflect sustained clinical benefit. Where possible, we have distinguished findings between exclusive EC users, dual users, and former smokers, observing that dual users generally exhibited more adverse profiles. Additionally, we now provide brief descriptions of key biomarkers: hs-CRP and IL-6 as markers of systemic inflammation, MDA as an oxidative stress indicator, and FMD as a vascular function measure - all relevant to cardiovascular risk but affecting different physiological pathways. [40]. The reviewed studies demonstrated a medium impact on the vascular function, comprising the endothelial dysfunction and PWV as the prevalent markers of the cardiovascular risk. Such findings correspond with the earlier literature, which has proposed a similar direction of effects with the ECs, although the extent of these effects differs among studies [41].

When compared to conventional smoking, the use of ECs was linked to a lesser extent of cardiovascular damage, although it nevertheless led to a significant degree of alterations in vascular stiffness and oxidative stress [42]. As an illustration, Fetterman et al. [26] and Ikonomidis et al. [23] identified that the use of ECs and smoking led to higher vascular dysfunction, but the effect of ECs derivation on PWV and FMD was slightly less than that of CC. The results align with those of the investigation by George et al. [25], which proposed that the substitution of smoking with the use of ECs might lead to the enhancement of vascular health, especially in the short-term perspective [43].

Nevertheless, the effect of EC on biomarkers for oxidative stress and inflammation in this study remained high, although less pronounced than in smokers. The research of Shahab et al. [22] and Christensen et al. [27] confirms the observation that ECs are responsible for other elevated levels of F2-isoprostane, a biomarker of oxidative stress, and of inflammation in comparison to nonsmokers [44]. The implication is that although ECs might lower exposure to some harmful chemicals, they present their own cardiovascular hazards.

The impact of the cardiovascular effects of EC, specifically among the dual users who utilize both smoking and EC, is still an issue. The heterogeneity in the effects observed between studies, as indicated by high heterogeneity in the meta-analysis, is probably caused by the variations in the study design, population characteristics, and duration of intervention. These variations highlight the complexity of comprehending the complete cardiovascular effect of EC usage and the necessity of additional clarification regarding long-term impacts, especially in dual users or ex-smokers who switch to ECs [45].

Limitations

This systematic review does offer useful knowledge of EC use and its cardiovascular impacts. There are a number of limitations that should be discussed, however. First, numerous studies with fairly modest sample sizes were included in the review. These smaller sample sizes have more variability, and this may make the results lean in one direction or another and add to the uncertainty with respect to the effect sizes. Moreover, heterogeneity between studies was significantly large, with an I² value reflecting large variability (as high as 99%). This indicates that variability in populations of participants, study design, and outcomes assessed could have been responsible for the observed heterogeneity. The additional fact that studies used different methodologies, e.g., differing definitions of EC use and intervention duration, adds to the complexity in comparing findings. The studies also varied in terms of the cardiovascular outcomes they were measuring, with some on vascular function, others on inflammatory biomarkers, and a few on symptom-based events such as MI. Such heterogeneity prevents drawing conclusions regarding the long-term effects of EC use on CVD. Finally, most of these studies were of fairly short duration, so our understanding of the long-term cardiovascular risks of EC use is still limited.

Future Research

Future analysis is crucial for better understanding the cardiovascular effects of EC use and addressing gaps highlighted in this review. Large-scale studies using mixed populations need to be conducted to increase the generalizability of results. Older people and those with pre-existing CVD should be studied in these investigations since their risk may not be the same as that of healthy young adults. Long-term studies are specifically valuable, stretching the evaluation of EC exposure over a period of several years to reveal possible long-term effects on cardiovascular well-being. Use of standardized methods to assess cardiovascular outcomes vascular function, oxidative stress, and inflammation will be critical for maintaining consistency between studies and enabling meaningful comparisons. Furthermore, investigating the implications of ECs with no nicotine is also paramount since most studies today target products with nicotine. Doing so will enable researchers to know if nicotine is responsible for most cardiovascular hazards or if the device is largely contributing to these risks. Lastly, RCTs with robust randomization and blinding strategies must be used to reduce bias and offer more credible data.

## Conclusions

This meta-analysis and systematic review offer a critical comparison between EC utilization and conventional cigarette smoking and nonsmoking on cardiovascular outcomes. The results elucidate that although ECs have fewer cardiovascular risks than traditional cigarettes, they are still responsible for vascular dysfunction, oxidative stress, as well as possible cardiovascular events. The impact on biomarkers like PWV and endothelial function also has high to moderate heterogeneity between studies, indicating that the effect of EC on cardiovascular condition remains unclear and could be contingent upon variables like the level of nicotine, exposure duration, and design of individual studies. Although EC could be an aid for dangerous reduction of smoking cessation, particularly when used to substitute CC, they are not risk-free. The available evidence suggests that utilizing both EC and traditional cigarettes may worsen cardiovascular risks, making the prevention of concurrent use essential. In spite of the overall optimistic perception of EC as a smoking substitute, the heterogeneity between studies and the short-term design of most research highlight the necessity for longer-term research capable of capturing more precisely the long-term impacts on cardiovascular morbidity. In addition, there is a need for standardization of study designs and outcomes to improve comparability and consistency of future research.
